# Supplementary material for: Neural networks to learn protein sequence–function relationships from deep mutational scanning data
Source: Proc Natl Acad Sci U S A. 2021 Nov 23;118(48):e2104878118. doi: 10.1073/pnas.2104878118 (PMC8640744; doi:10.1073/pnas.2104878118)
Supplement: Supplementary File [file pnas.2104878118.sapp.pdf]

PNAS
www.pnas.org

Supplementary Information for

Neural networks to learn protein sequence-function relationships from deep mutational scanning data

Sam Gelman, Sarah A. Fahlberg, Pete Heinzelman, Philip A. Romero, and Anthony Gitter

Anthony Gitter
E-mail: gitter@biostat.wisc.edu

This PDF file includes:

Supplementary text
Figs. S1 to S12
Tables S1 to S5
SI References

Supporting Information Text

Methods

Designed GB1 variant gene synthesis and protein expression. We designed the genes encoding the designed GB1 variants by making codon substitutions into the base wild-type GB1 gene sequence. If there were multiple codon options for an amino acid, we chose the particular codon randomly from a set of 31 codons that are optimized for expression in E. coli (1). For our expression construct, we included an upstream bicistronic design (BCD) element to minimize any influence of mRNA secondary structure on protein expression (2) and also included an N-terminal 6x His-tag with a five-amino-acid linker for protein purification. We ordered wild-type GB1 and the five designed GB1 variants from Twist Biosciences cloned into the pET21(+) protein expression vector.

We expressed wild-type GB1 and the five designed GB1 variants using a standard T7 expression system. We transformed the six plasmids into BL21(DE3) E. coli cells. We expressed the GB1 variants by inoculating LB cultures containing 100 μg/mL carbenicillin with a 1:100 dilution of overnight cultures, incubating these cultures shaking at 37°C until they reached an OD600 of 0.4-0.6, and inducing with 400 μM Isopropyl β-D-1-thiogalactopyranoside (IPTG). We then incubated these expression cultures overnight at 20°C while shaking, pelleted the cells by centrifuging at 3000 g for 20 minutes at 4°C, and stored the cell pellets at -80°C.

We determined the level of soluble protein expression using sodium dodecyl sulphate–polyacrylamide gel electrophoresis (SDS-PAGE). We thawed the cell pellets on ice and resuspended into 0.5 mL of Buffer A (20 mM sodium phosphate pH 7.3, 500 mM NaCl, 20 mM imidazole). We then added 2.5 mL of lysis buffer (Buffer A + 0.60x BugBuster + 2 U/mL DNaseI (Thermo Fischer) + 1 mg/mL hen egg white lysozyme) to each sample and incubated at room temperatures for 5 minutes to yield the total cell lysate. We obtained the soluble protein fraction by centrifuging at 21,000 g for 70 minutes and extracting the supernatant. We then ran samples of the total cell lysate and soluble fractions on a Novex 4-20% Tris-Glycine SDS-PAGE gel (Thermo Fischer). After staining, we analyzed the gels to qualitatively evaluate whether the expressed proteins were present in the soluble fraction

Protein purification and circular dichroism spectroscopy. We expressed wild-type GB1 and Design10 using the above protocol, with the exception that the expression cultures were incubated at 16°C for 24 hours. We thawed the cell pellets on ice, resuspended in 2.5 mL of Buffer A, sonicated for 1 minute with 5 second pulses spaced by 15 second resting periods, and centrifuged for 10 minutes at 21,000 g to obtain the soluble protein fraction. We then ran the soluble fraction over a Ni Sepharose 6 Fast Flow column (Cytiva Life Sciences) that was equilibrated with Buffer A, washed with 3 column volumes of Buffer A, and eluted in 1.5 mL fractions of Buffer B (20 mM sodium phosphate pH 7.3, 500 mM NaCl, 500 mM imidazole). We ran the elution fractions over SDS-PAGE and pooled the fractions that contained the target protein. Finally, we aliquoted the purified protein, flash froze the aliquots in liquid nitrogen, and stored at -80°C.

For circular dichoism (CD) spectroscopy, we thawed the purified protein samples on ice and dialyzed overnight in 20 mM sodium phosphate pH 8.0 at 4°C to remove imidazole. We then determined the protein concentrations using a Nanodrop spectrophotometer. The CD measurements were then performed by UW-Madison's Biophysics Instrumentation Facility. They measured CD spectra using a 1 mm pathlength on an AVIV Model 420 Circular Dichroism Spectrometer at 4°C. The CD spectra for Design10 was normalized to the wild-type GB1 spectra at 222 nm.

GB1 yeast display plasmid construction and flow cytometric IgG binding affinity titration. We synthesized wild-type and Design10 variant GB1 genes as yeast codon-optimized gBlocks (Integrated DNA Technologies, Coralville, IA). The gBlocks were ligated into the unique NheI and BamHI sites of the yeast surface display vector pCTCON2 (provided by Dane Wittrup, MIT). We synthesized A24Y and E19Q+A24Y variant GB1 genes as yeast-optimized gene fragments (Twist, San Francisco, CA). The gene fragments were ligated into a golden-gate compatible version of pCTCON2 at the NheI, BamHI sites. This yeast display vector fuses the Aga2p protein to the N-terminus of GB1.

We transformed plasmid DNA into yeast display *Saccharomyces cerevisiae* strain EBY100 made competent using the Zymo Research Frozen EZ Yeast Transformation II kit with transformants grown on synthetic dropout (SD) -Trp (MP Biomedicals, Irvine, CA) agar plates for two days at 30°C. After two days, individual colonies were picked into 4 mL of low-pH Sabouraud Dextrose Casamino Acid media (20 g/L dextrose, 6.7 g/L yeast nitrogen base, 5 g/L casamino acids, 10.4 g/L sodium citrate, 7.4 g/L citric acid monohydrate) and grown overnight at 30°C and 250 rpm. For induction of GB1 display, we started a 5 mL Sabouraud Galactose Casamino Acid (8.6 g/L $NaH_2PO{*}H_2O$, 5.4 g/L Na_2HPO_4, 20 g/L galactose, 6.7 g/L yeast nitrogen base, 5 g/L casamino acids) culture at an optical density, as measured at 600 nm, of 0.5 and shook overnight at 250 rpm and 20°C.

We harvested approximately 2×10^5 yeast cells for each titration data point by centrifugation after overnight incubation, washed them once in pH 7.4 Phosphate Buffered Saline (PBS) containing 0.2% (w/v) bovine serum albumin (BSA), and incubated them overnight at 4°C on a tube rotator at 18 rpm in between 100 μL and 800 μL of PBS/0.2% BSA containing various concentrations of mouse IgG2a (BioLegend, San Diego, CA) that had been conjugated with Alexa647 using NHS chemistry (Molecular Probes, Eugene, OR). Volumes of Alexa647 IgG-containing incubation solution were varied to prevent ligand depletion from occurring in the lowest IgG concentration incubation tubes. Following overnight incubation, yeast were washed once in PBS/0.2% BSA and resuspended in ice cold PBS for flow cytometric analysis. Analyses were performed using a Fortessa analyzer (Becton Dickinson), and the mean of the fluorescence distribution was reported.

We performed duplicate fluorescence measurements for all nine IgG concentrations tested. We then fit a Hill function to the average of these duplicate measurements. We were able to determine the K_d of Design10 as 5 nM because the binding curve

was beginning to display saturation. We were unable to determine the K_d of wild-type, A24Y, or E19Q+A24Y GB1 variants because the proteins were less than 50% bound at the highest IgG concentration tested.

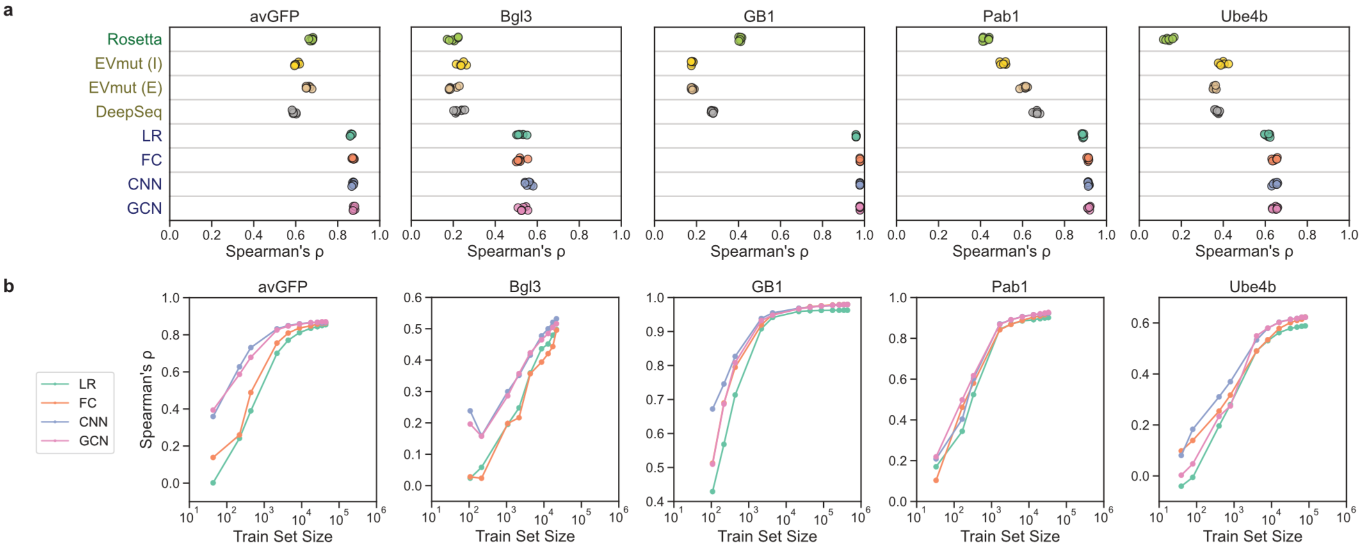

Fig. S1. Model evaluation using Spearman's correlation coefficient. (a) Spearman's correlation coefficient between true and predicted scores for Rosetta, EVmutation, DeepSequence, linear regression (LR), fully connected network (FC), sequence convolutional network (CNN), and graph convolutional network (GCN). EVmutation (I) refers to the independent formulation of the model that does not include pairwise interactions. EVmutation (E) refers to the epistatic formulation of the model that does include pairwise interactions. Each point corresponds to one of seven random train-tune-test splits. (b) Spearman's correlation performance of supervised models trained with reduced training set sizes.

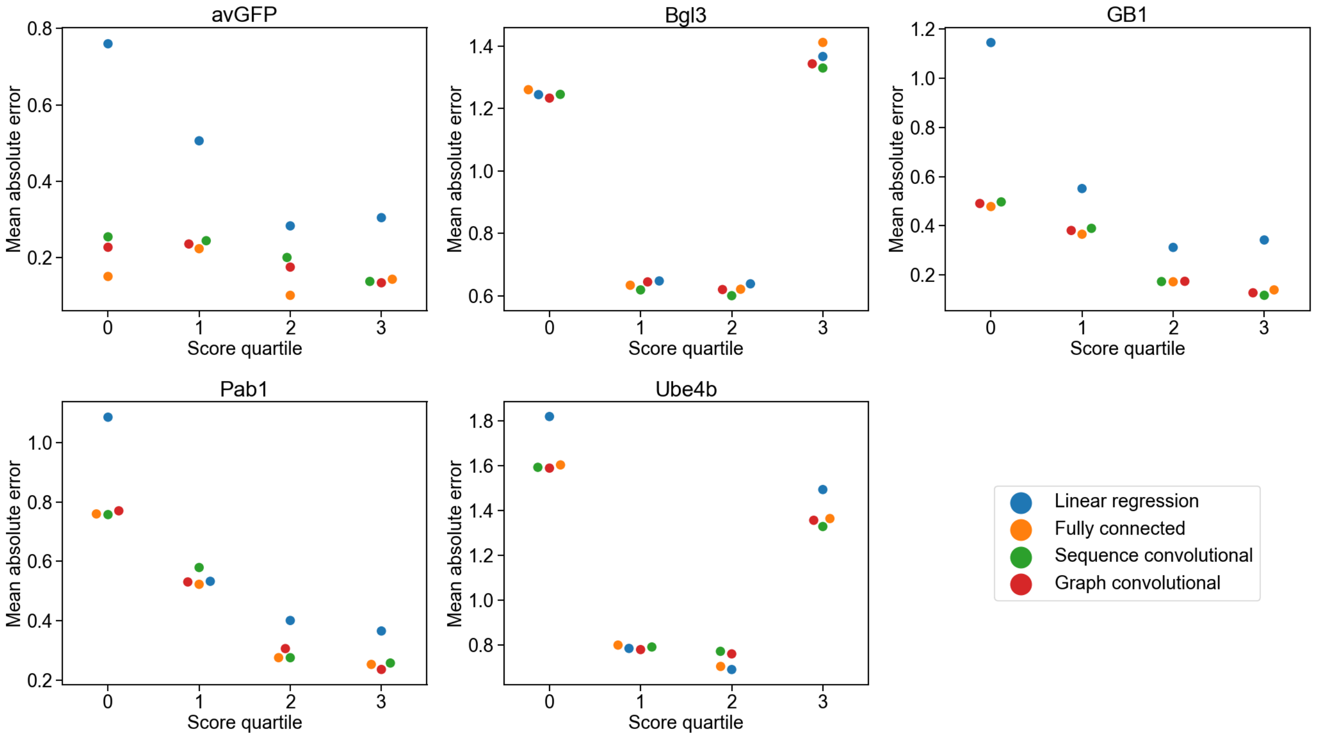

Fig. S2. Mean absolute error vs. score quartile. The mean absolute error in the models' predictions grouped by score quartile. Linear regression has a substantial jump in error for low-scoring variants compared to the other models in avGFP, GB1, Pab1, and Ube4b.

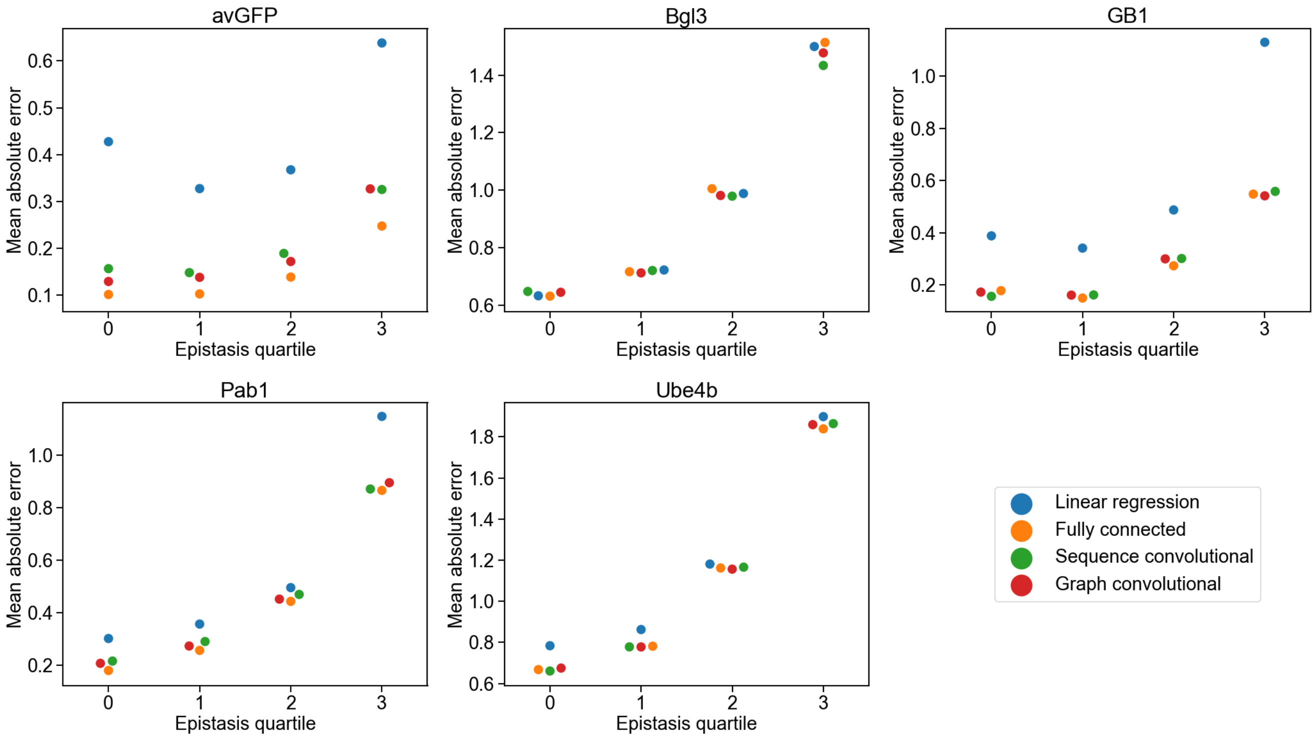

Fig. S3. Mean absolute error vs. epistasis quartile. The mean absolute error in the models' predictions grouped by absolute epistasis quartile. We compute epistasis by subtracting the expected score for the multi-mutant sequence from the true score. The expected score for the multi-mutant sequence is the sum of the corresponding single-mutant scores, truncated to the observed minimum or maximum in the dataset. Linear regression has a substantial jump in error for high-epistasis variants compared to the other models in avGFP, GB1, and Pab1.

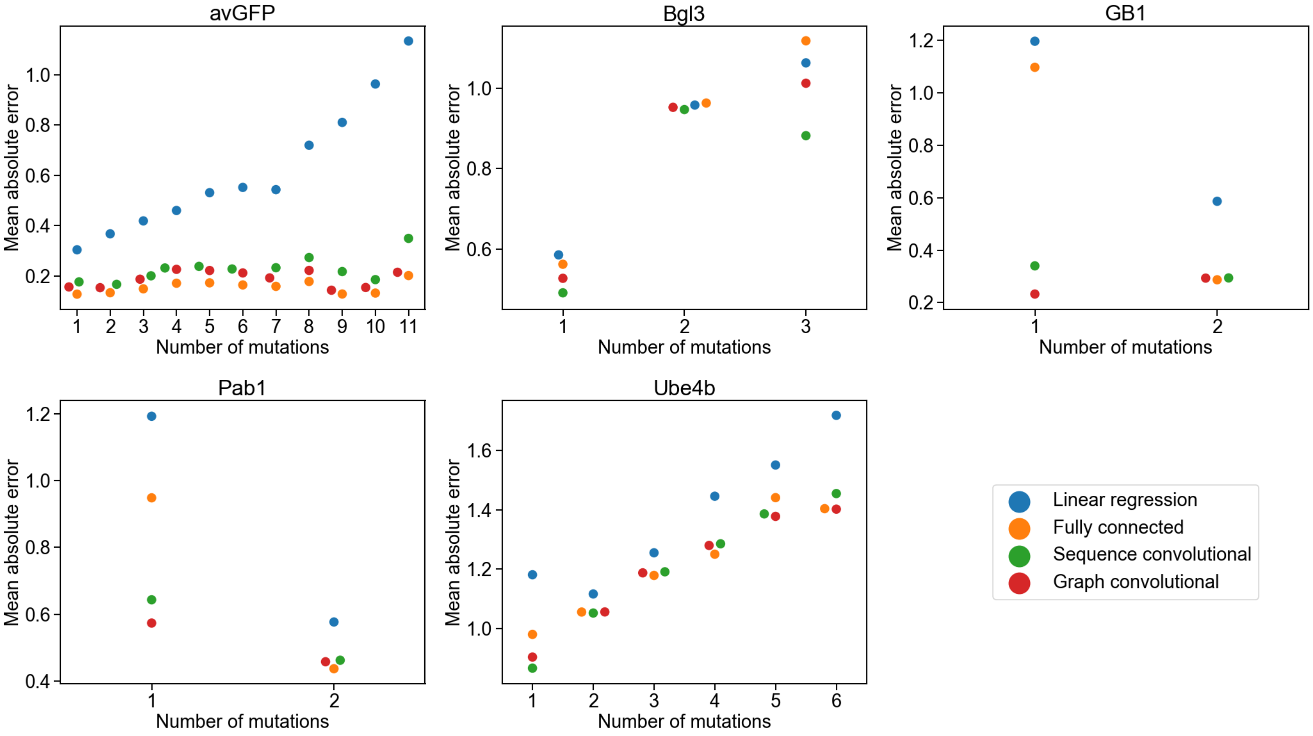

Fig. S4. Mean absolute error vs. number of mutations. The absolute error in the models' predictions for each variant grouped by the number of mutations in the variant. Linear regression struggles with increasing numbers of mutations in avGFP. The convolutional networks perform better than linear regression and the fully connected network on single-mutation variants in GB1 and Pab1.

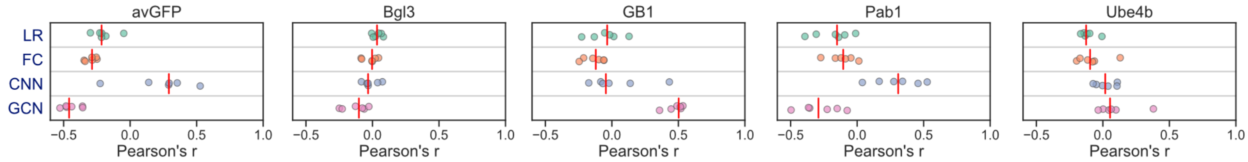

Fig. S5. Positional extrapolation. Model performance when making predictions for variants containing mutations in positions that were unmodified in the training data (positional extrapolation). Each point corresponds to one of six replicates, and the red vertical line denotes the median.

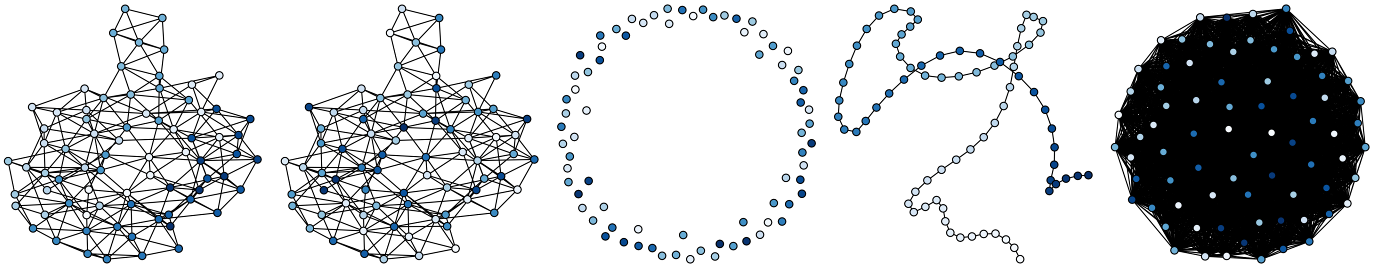

Fig. S6. Protein structure graphs for Pab1. The graph convolutional network uses a graph of the protein's structure to determine which residues are close together. In addition to the standard graph based on the protein's actual structure, we tested four baseline graphs: a shuffled graph based on the standard graph but with shuffled node labels, a disconnected graph with no edges, a sequential graph containing only edges between sequential residues, and a complete graph containing all possible edges. The graphs pictured are for the Pab1 dataset. The structured graph uses a distance threshold of 7Å to determine which residues should be connected with edges (selected by hyperparameter sweep). The nodes are colored according to each residue's sequence position, with light colors corresponding to residues at the start of the sequence and dark blue colors corresponding to residues at the end of the sequence.

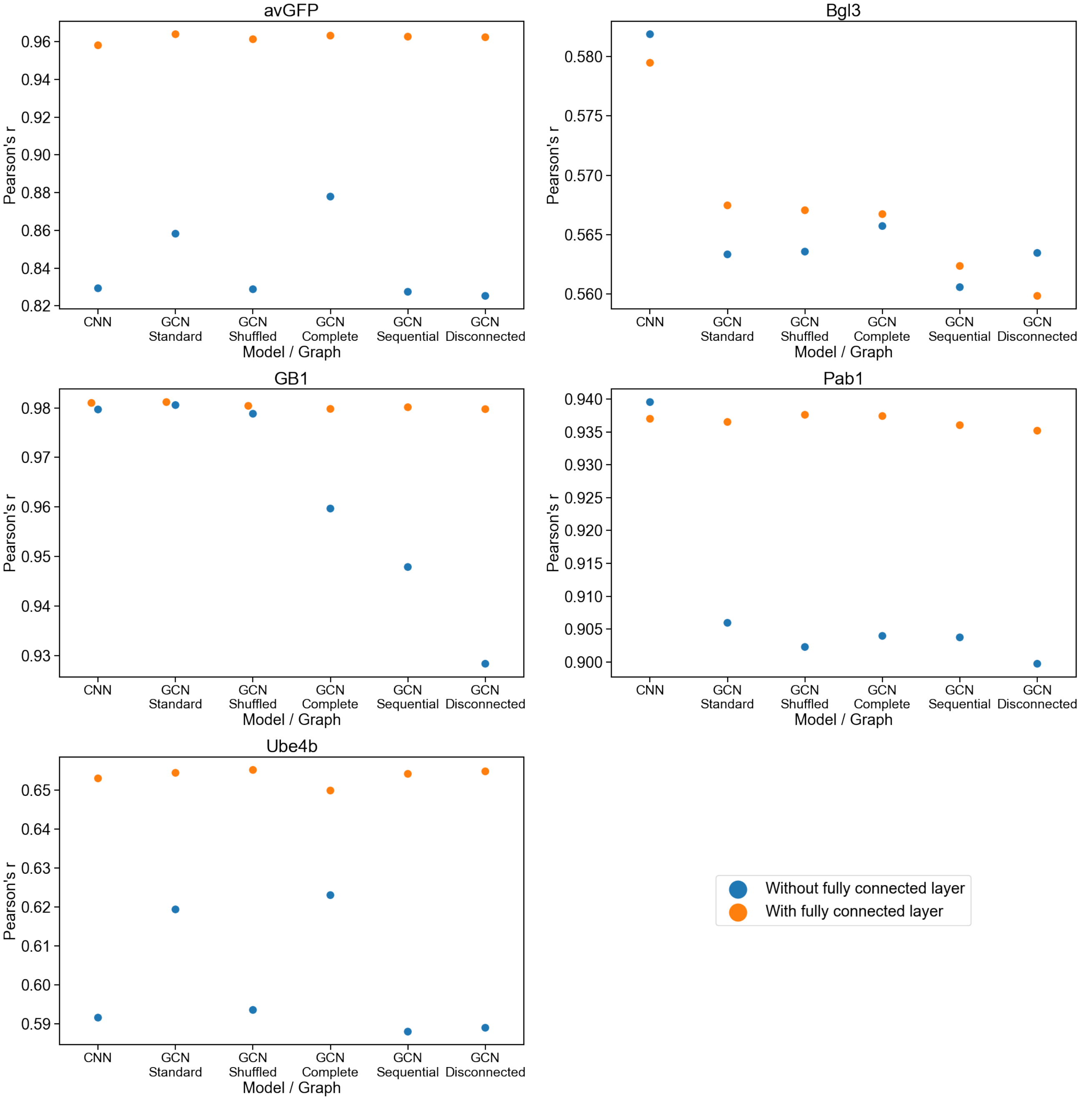

Fig. S7. Convolutional networks with and without a fully connected layer. The correlation performance of sequence convolutional and graph convolutional networks trained with various baseline structure graphs, with and without a final fully connected layer. The standard graph is based on the protein's actual structure. The shuffled graph is a version of the regular structured graph with shuffled node labels. The complete graph contains all possible edges between residues. The sequential graph only contains edges between sequential residues. The disconnected graph contains no edges. The fully connected layer at the end of the network compensates for apparent differences in performance caused by type of convolutional network or different graph structures.

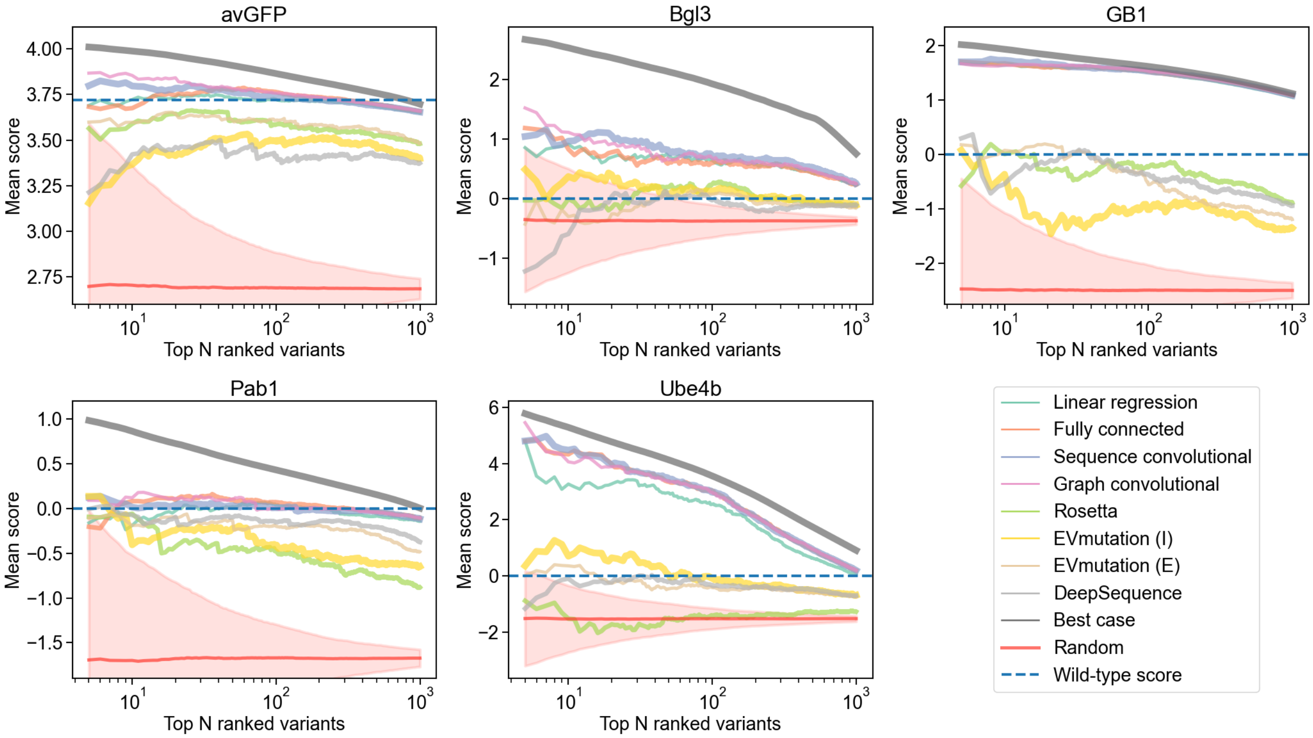

Fig. S8. Mean score of highest ranked variants. The mean score of each model's ranking of the highest scoring test set variants. For the most part, the supervised models prioritize variants whose average score is higher than the wild-type. The random baseline is shown with the mean and 95% confidence interval.

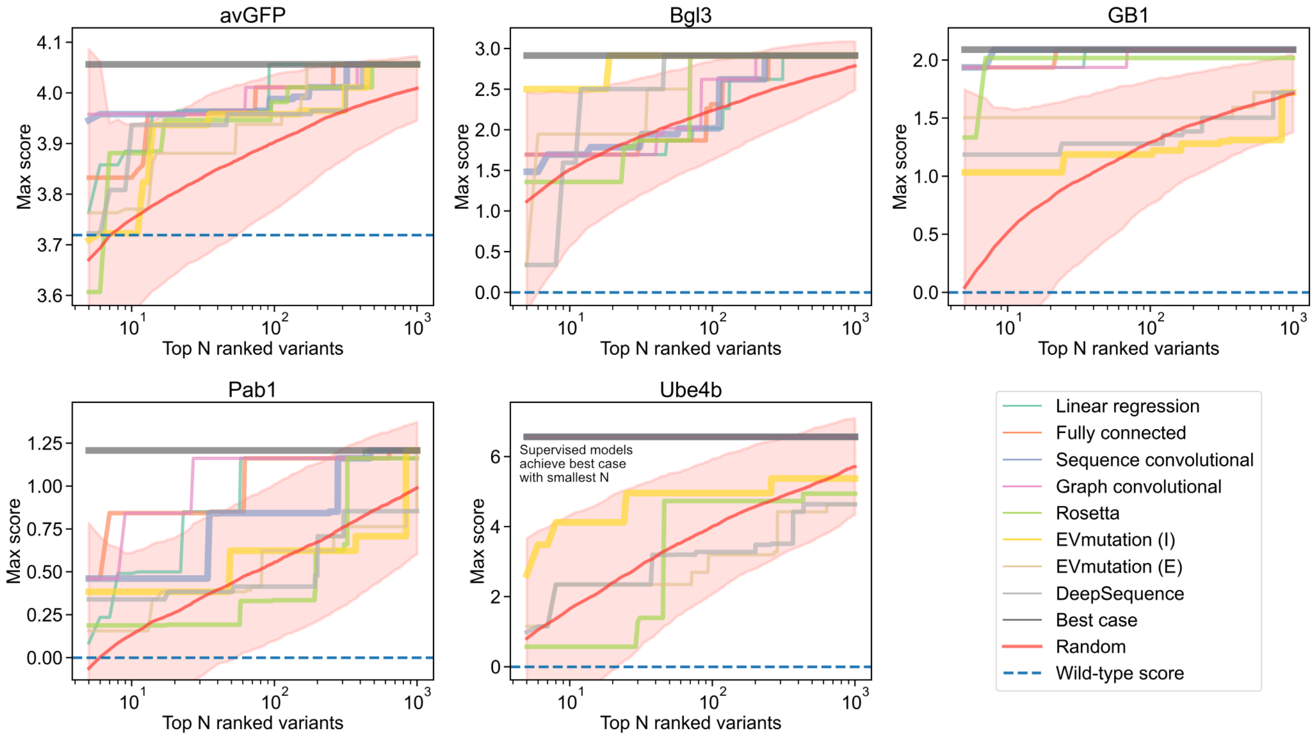

Fig. S9. Max score of highest ranked variants. The max score in each model's ranking of the highest scoring test set variants. For Ube4b, the supervised models prioritize a variant with the true max score with the smallest tested budget (N=5), thus all the lines corresponding to the supervised models are hidden behind the line for the true score. Nearly all models across all datasets prioritize variants whose max score is higher than the wild-type. The random baseline is shown with the mean and 95% confidence interval.

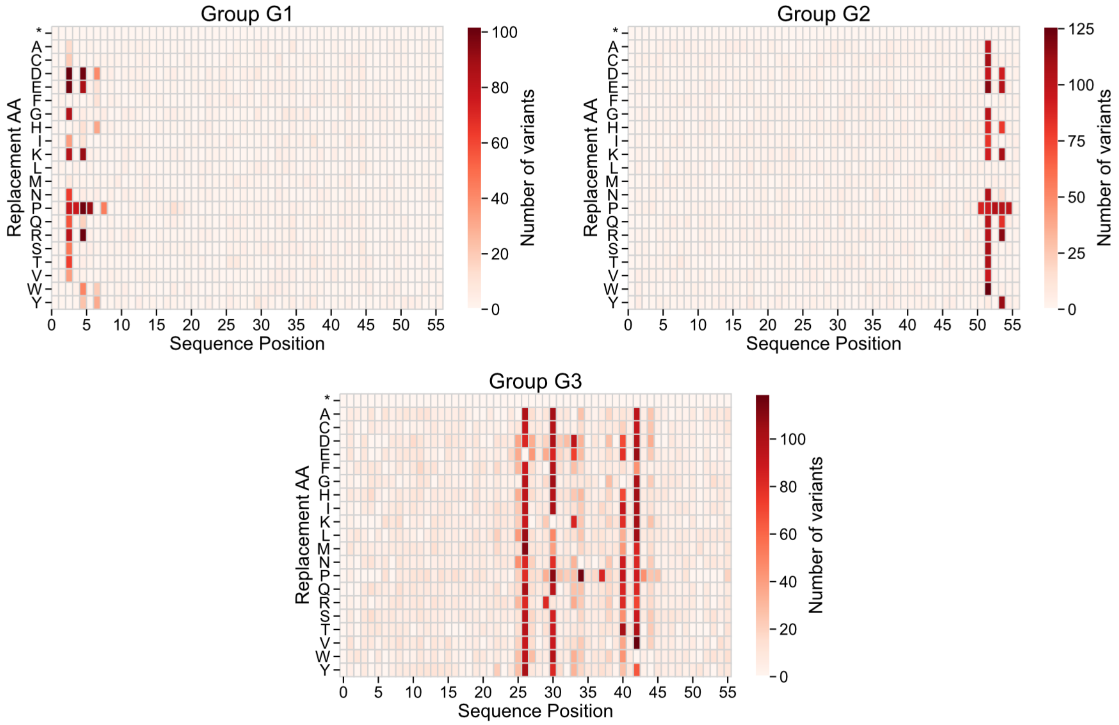

Fig. S10. Mutations in GB1 latent space groups. Heat maps showing the number of occurrences of mutations for each annotated group in the GB1 latent space in Figure 4a. Groups G1 and G2 contain variants with mutations at core residues near the start and end of the sequence, respectively. Group G3 contains variants with mutations at surface interface residues.

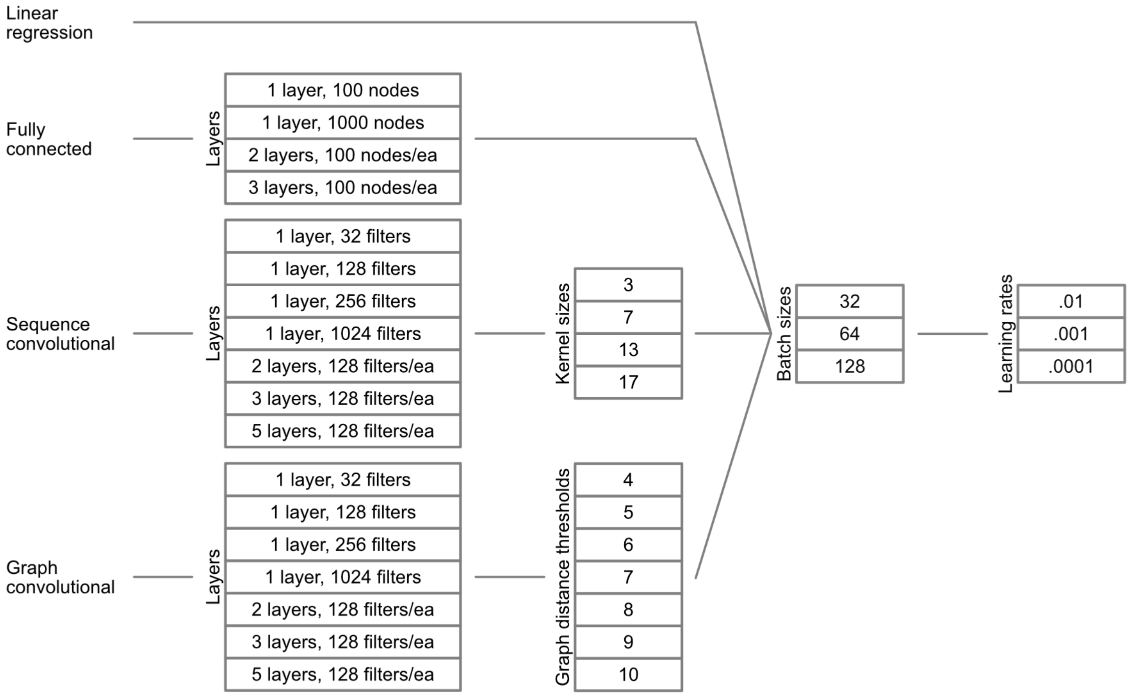

Fig. S11. Hyperparameter sweep. We performed an exhaustive hyperparameter sweep for each dataset and type of model using all possible combinations of these hyperparameters.

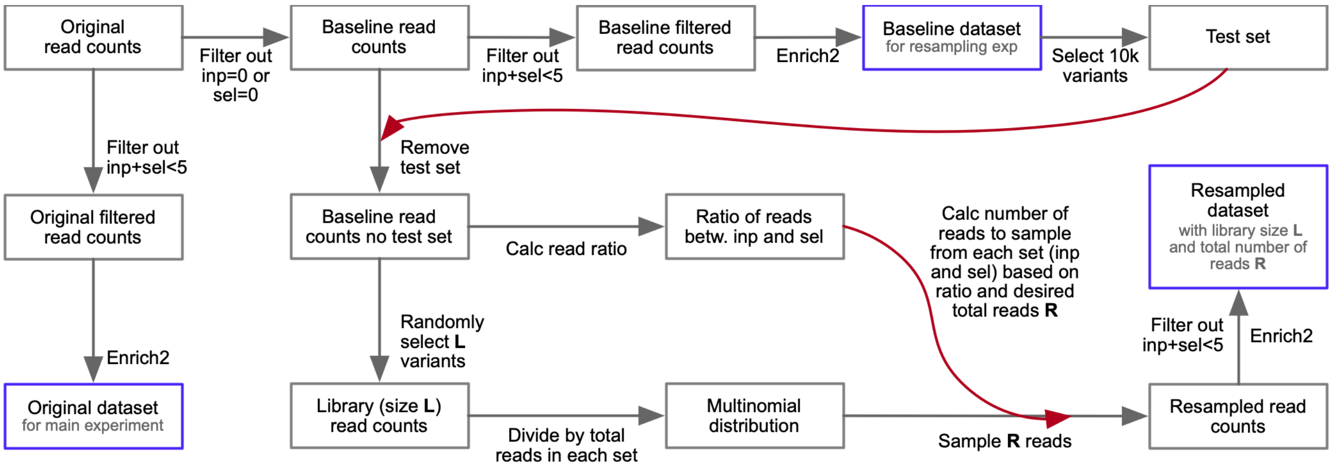

Fig. S12. Generation of resampled GB1 datasets. Flowchart showing how we created resampled GB1 datasets corresponding to different library sizes and numbers of reads.

Table S1. Designed GB1 sequences.

Variant	Amino acid sequence
Wild-type	MQYKLILNGKTLKGETTTEAVDAATAEKVFKQYANDNGVDGEWTYDDATKTFTVTE
Design10	MQYKLILNGKTLKGETWTWGHDPYRAEKKFKLYANDNGVWGEWTYDDATKTFTVTE
Design20	MQYKLEANWKTLKGETFTIAVDDYRAEKHFKLMMNANNIYGLWTYDRATKTFGMTE
Design30	MQYKLETWHPWNAGERNRVAVVAYMAEKNFKNKLNANNWWGTWTIDWAGKTFGCTA
Design40	MAFKNEAWWPWWCEEINRVAHAAWWAEVNFKNKLNANNWFGCWADCWAHGIFGATT
Design50	MPHTCEANDWWNWEVVNWSRHAPYRAEIHHKNEAFSLNWLGTWQGIRVQDRFNFGT

The GB1 wild-type sequence and the designed sequences with increasing numbers of mutations (10, 20, 30, 40, and 50) from wild-type.

Table S2. Diversity in designed GB1 sequences.

Mutation	Frequency	Present in Design10
A24Y	0.93	Y
D40W	0.52	Y
D40Y	0.48	N
V29K	0.48	Y
Q32L	0.45	Y
E42Q	0.42	N
A34M	0.33	N

We repeated our hill climbing protein design approach 100 times to generate 100 sequences with 10 mutations each. We found 27 of 100 design runs converged to the same sequence. The other 73 represent distinct local optima in the landscape. A number of mutations were observed across multiple designs, and some of these were present in Design10. This table lists mutations common across the designs and their frequencies.

Table S3. Selected hyperparameters.

Dataset	Model type	Key parts of architecture	Learning rate	Batch size	Epochs
avGFP	Linear regression	Linear regression	0.0001	128	90
	Fully connected	3 layers, 100 hidden units each	0.0001	32	134
	Sequence convolutional	5 layers, kernel size 3, 128 filters	0.001	64	113
	Graph convolutional	2 layers, 7Å threshold, 128 filters	0.0001	32	130
Bgl3	Linear regression	Linear regression	0.0001	128	164
	Fully connected	2 layers, 100 hidden units each	0.0001	32	187
	Sequence convolutional	1 layer, kernel size 17, 32 filters	0.0001	64	102
	Graph convolutional	1 layer, 6Å threshold, 32 filters	0.0001	128	129
GB1	Linear regression	Linear regression	0.0001	128	27
	Fully connected	1 layer, 1000 hidden units	0.0001	64	110
	Sequence convolutional	3 layers, kernel size 17, 128 filters	0.0001	32	27
	Graph convolutional	5 layers, 7Å threshold, 128 filters	0.0001	32	109
Pab1	Linear regression	Linear regression	0.001	128	47
	Fully connected	3 layers, 100 hidden units each	0.001	128	108
	Sequence convolutional	3 layers, kernel size 17, 128 filters	0.0001	128	42
	Graph convolutional	1 layer, 7Å threshold, 32 filters	0.0001	128	232
Ube4b	Linear regression	Linear regression	0.0001	64	73
	Fully connected	3 layers, 100 hidden units each	0.0001	64	124
	Sequence convolutional	5 layers, kernel size 3, 128 filters	0.0001	128	29
	Graph convolutional	3 layers, 7Å threshold, 128 filters	0.0001	128	92

The hyperparameters selected by a hyperparameter sweep for the main experiment. There are additional parts of the architecture that were not part of the hyperparameter sweep. For example, the fully connected networks have a dropout layer after every dense layer. The convolutional networks have a dense layer and a dropout layer before the output node. Experiments with reduced training set sizes and GB1 resampling used the same architectures selected for the main experiment, but they had their own sweeps for learning rate and batch size.

Table S4. Numbers of trainable parameters.

Model	avGFP	Bgl3	GB1	Pab1	Ube4b
Linear regression	9,481	20,041	2,241	3,001	4,081
Fully connected	968,401	2,014,301	2,242,001	320,401	428,401
Sequence convolutional	3,118,409	1,573,993	747,081	990,281	1,390,409
Graph convolutional	3,077,065	1,605,993	858,953	242,793	1,381,961

The number of trainable parameters in each model.

Table S5. Main software packages

Library	Version
python	3.6.8
cudatoolkit	10.0.130
cudnn	7.6.0
tensorflow-gpu	1.14.0
gast	0.2.2
numpy	1.16.4
joblib	0.13.2
matplotlib	3.1.1
networkx	2.3
pandas	0.25.0
scikit-learn	0.21.2
scipy	1.3.0
seaborn	0.9.0
enrich2	1.2.1

The main libraries and version numbers used to train and evaluate models.

References

1. G Boël, et al., Codon influence on protein expression in E. coli correlates with mRNA levels. *Nature* **529**, 358–363 (2016).
2. VK Mutalik, et al., Precise and reliable gene expression via standard transcription and translation initiation elements. *Nat. Methods* **10**, 354–360 (2013).
